# Phenylhexyl isothiocyanate has dual function as histone deacetylase inhibitor and hypomethylating agent and can inhibit myeloma cell growth by targeting critical pathways

**DOI:** 10.1186/1756-8722-1-6

**Published:** 2008-06-09

**Authors:** Quanyi Lu, Xianghua Lin, Jean Feng, Xiangmin Zhao, Ruth Gallagher, Marietta Y Lee, Jen-Wei Chiao, Delong Liu

**Affiliations:** 1 Division of Hematology/Oncology, New York Medical College, Valhalla, NY 10595, USA; 2 Department of Hematology, Zhongshan Hospital of Xiamen University, Xiamen, Fujian Province, PR China; 3 Department of Biochemistry and Molecular Biology, New York Medical College, Valhalla, NY 10595, USA

## Abstract

Histone deacetylase (HDAC) inhibitors are a new class of chemotherapeutic agents. Our laboratory has recently reported that phenylhexyl isothiocyanate (PHI), a synthetic isothiocyanate, is an inhibitor of HDAC. In this study we examined whether PHI is a hypomethylating agent and its effects on myeloma cells. RPMI8226, a myeloma cell line, was treated with PHI. PHI inhibited the proliferation of the myeloma cells and induced apoptosis in a concentration as low as 0.5 μM. Cell proliferation was reduced to 50% of control with PHI concentration of 0.5 μM. Cell cycle analysis revealed that PHI caused G1-phase arrest of RPMI8226 cells. PHI induced p16 hypomethylation in a concentration- dependent manner. PHI was further shown to induce histone H3 hyperacetylation in a concentration-dependent manner. It was also demonstrated that PHI inhibited IL-6 receptor expression and VEGF production in the RPMI8226 cells, and reactivated p21 expression. It was found that PHI induced apoptosis through disruption of mitochondrial membrane potential. For the first time we show that PHI can induce both p16 hypomethylation and histone H3 hyperacetylation. We conclude that PHI has dual epigenetic effects on p16 hypomethylation and histone hyperacetylation in myeloma cells and targets several critical processes of myeloma proliferation.

## Background

Despite many recent advances in treatment, multiple myeloma (MM) remains as an incurable disease without an allogeneic hematopoietic cell transplantation. The emergence of drug resistance and incomplete responses have been the major obstacles for improving the treatment results [1,2]. The new treatment strategies have been based largely upon targeting specific molecules or pathways, such as proteosome inhibitors and thalidomide analogs. Aberrant methylation of gene promoter regions is a widely studied epigenetic process in malignant disorders. Cell cycle inhibitors of p15 and p16 are the tumor suppressor genes frequently affected by this epigenetic change [3,4]. The aberrant methylation of gene promoter regions is associated with loss of gene function. In addition to gene deletions and mutations, quantitative changes in gene methylation status play a significant role in tumorigenesis [5]. Hypermethylation of p15 and p16 promoter CpG islands has been reported in MM clinical specimens and myeloma cell lines [4,6,7]. The methylation status of p15 and p16 genes were not significantly different between MM and MGUS (monoclonal gammopathy of unknown significance) nor in pre-treated and post-treated patients with MM [6-8]. It was further demonstrated in MM patients that p16 hypermethylation is associated with high plasma cell proliferation, higher β_2_-microglobulin concentration, and shorter survival, whereas no such clear correlation was found with p15 CpG island hypermethylation [4,7,9].

The proliferation and survival of myeloma cells are also potentiated by IL-6 and IL-6 receptor signal transduction through autocrine and paracrine stimulation [10,11]. Exogenous IL-6 was able to block the apoptosis induced by the chemotherapeutic agent dexamethasone [10,12]. Increased angiogenesis and microvascular density in the bone marrow microenvironment correlate with poor prognosis and drug resistance of myeloma cells [13-15]. Cytokines that augment angiogenesis are known to be present at elevated levels in the bone marrow. The vascular endothelial growth factor (VEGF) is one of those elevated cytokines associated with angiogenesis. Thalidomide and its derivative, lenalidomide (CC-5013, Revlimid; Celgene), are inhibitors of angiogenesis and are widely used for MM therapy [1].

In the search for novel molecular targets, histone deacetylases (HDACs) that affect epigenetic processes have emerged as one of the potential targets [16,17]. Recent studies have indicated that the expression of various genes that regulate differentiation, proliferation, and apoptosis are also influenced by HDACs. Aberrant histone acetylation appears to play an important role in the development of numerous malignancies [18,19]. Agents that modify histone acetylation thus show great promise against various malignancies [20-26]. Vorinostat (Suberoylanilide hydroxamic acid, SAHA, Zolinza; Merck) is among the first HDAC inhibitors approved for clinical treatment of cutaneous T cell lymphoma [27,28]. Our laboratory has recently reported that a synthetic isothiocyanate, phenylhexyl isothiocyanate (PHI), is an inhibitor of HDACs [29,30]. We have found that PHI can induce selective histone acetylation and lead to cell cycle arrest and apoptosis in human leukemia cells and prostate cancer cells [29-31]. Oral feeding of PHI to immunodeficient mice inhibited the tumorigenesis of human leukemia cells in vivo [29,30]. We have further demonstrated that PHI has a selective effect in inducing apoptosis in cancer cells, but not in normal cells [29-31]. In this study we demonstrated, for the first time, that PHI has dual epigenetic effects of causing histone hyperacetylation and p16 hypomethylation in multiple myeloma cell line RPMI8226.

## Methods

### Cell culture and chemicals

The preparation of PHI has been described previously [29,30]. Human myeloma cell line RPMI 8226 was obtained from American Type Culture Collection (ATCC, Manassas, VA). Cells were seeded at 0.3 × 10^6 ^per ml of RPMI-1640 medium, supplemented with 10% heat-inactivated fetal calf serum, 100 IU penicillin/ml and 100 ug streptomycin/ml, and maintained at 37°C in a humidified atmosphere containing 5% CO_2_. Cells in exponential growth were exposed to PHI at various concentrations prepared in 75% methanol and PBS [29]. The control cultures were supplemented with the methanol-containing medium. Cell viability was determined from at least triplicate cultures by trypan blue exclusion method. Cell density was calculated by the viable cell counts per ml.

### Methylation specific PCR

Methylation specific PCR (MS-PCR) was performed using the procedure previously described [32]. RPMI 8226 cells at exponential growth were treated without or with PHI or Decitabine at various concentrations for 10 days. The DNA from the cells was extracted and bisulfite-converted for MS-PCR analyses. The primers for the methylated form of p16 are ttattagagggtggggcggatcgc (sense) and gaccccgaaccgcgaccgtaa (antisense). The primers for unmethylated form are ttattagagggtggggtggattgt (sense) and caaccccaaaccacaaccataa (antisense). The methylated PCR product covers 151 bp extending from bp 167 to bp 317, and the unmethylated product are 152 bp extending from bp 167 to bp 318 [33]. The amplification was performed in a thermocycler unit under the program conditions (Hotstart Kit, Quiaqen) as follows: 95°C for 15 min; then 40 cycles of 95°C for 15 sec, 60°C for 30 sec, 72°C for 30 sec; and finally 10 min at 72°C. At least two independent PCR amplifications were performed for each sample.

### Cell cycle and vascular endothelial growth factor (VEGF) measurements

Analysis of cell cycle phases was performed using a Becton-Dickinson FACScan flow cytometer according to the methods described previously [29] The cells were stained with propium iodide solution (50 μg/ml) on ice, and at least 10,000 cells were analyzed. For VEGF measurement, the RPMI 8226 cells were plated at 0.3 × 10^6 ^cells/ml, the cultures were incubated for designated time periods. The contents of VEGF in the culture supernatants were determined with a VEGF ELISA kit (R & D System, Minneapolis, MN, USA). The percent alteration of VEGF contents from PHI-exposed cells was calculated and compared to the control cultures.

### Measurements of mitochondrial membrane potential and apoptosis

The effects of PHI treatment on mitochondrial membrane potential was measured using a potential sensitive dye JC-1 (5,5',6,6'-tetrachloro-1,1',3,3'-tetraethylbenzimidazolylcarbocyanine iodide). The JC-1 dye bears a delocalized positive charge and enters the mitochondrial matrix due to the negative charge established by the intact mitochondrial membrane potential [34,35]. In healthy cells, JC-1 dye stains the mitochondria red due to formation of J-aggregates. In apoptotic cells, JC-1 dye accumulates in the cytoplasm in monomeric form (green fluorescence) due to collapse of the mitochondrial membrane potential. Stock solution of JC-1 (1 mg/ml) was prepared in DMSO and freshly diluted with the supplied assay buffer. RPMI8226 cells were incubated with medium containing JC-1 (10 μg/ml) for 15 min at 37°C. Cells were washed and re-suspended in 0.5 ml assay buffer and the fluorescence was measured using a Becton-Dickinson FACScan flow cytometer. Carbonyl cyanide 4-(trifluoromethoxy)phenylhydrazone (CCCP; 25 μM), an uncoupler of mitochondrial oxidative phosphorylation, was used as a positive control.

Apoptotic cells were determined by the characteristic morphology, and by the presence of DNA strand breaks detected with terminal deoxynucleotidyl transferase-mediated biotinylated UTP nick-end labeling (TUNEL). The TUNEL detection of apoptosis *in situ *was performed with a cell death detection kit (Roche Diagnostics, Indianapolis, IN). Briefly, cytospin preparations were fixed with 4% paraformaldehyde and incubated in Triton solution for 4 min on ice. Cells incubated with the solution lacking the terminal transferase were used as a negative control. The slides were counter stained with 5% methyl green and evaluated under a light microscope. The percentage of apoptotic cells was calculated by counting at least 500 cells from multiple fields.

### Western blot analysis

The expression levels of protein in RPMI 8226 were determined by Western blot analyses using standard procedures as described previously [29]. Total proteins were prepared from each culture condition with a lysis buffer containing freshly prepared protease inhibitors. The protein contents of the lysates were determined by using the BioRad Protein Assay kit (Bio Rad, Hercules, CA, USA) with a BSA standard. Proteins were subjected to SDS-PAGE, electrotransferred to nitrocellulose membrane, and immunoblotted with specific antibodies. Antibodies against acetyl-histone H3 were purchased from Upstate Biotechnology (Lake Placid, NY). Antibodies against p16 and IL-6R were purchased from Santa Cruz (Santa Cruz, CA, USA). β-actin was used as a loading control. Appropriate HRP- conjugated secondary antibodies were used. The reactive proteins were visualized using the ECL system.

### Statistical analysis

The data are presented as mean ± S.D. from multiple independent experiments. Results were evaluated by a two-sided paired Student's *t*-test for statistical difference.

## Results

### PHI inhibits proliferation and causes G1 arrest of multiple myeloma cells

**Figure 1:**
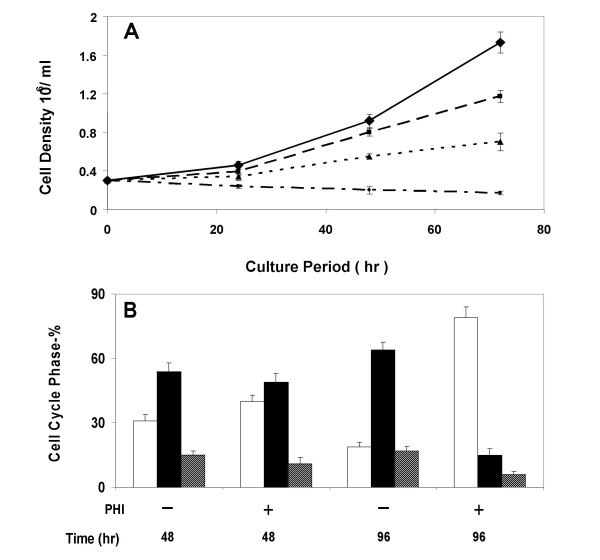
**PHI suppresses growth and causes cell cycle arrest of RPMI8226 myeloma cells**. (A). PHI suppresses growth of RPMI8226 myeloma cells. The myeloma cells were cultured with or without phenylhexyl isothiocyanate (PHI) at varying concentrations for 24, 48 or 72 hours. The cell number was recorded at each time point. Diamond (♦), control; Square (■), 0.1 μM; Triangle (), 0.5 μM; Dot (●); 5.0 μM. (B). PHI induces G1 growth arrest. The myeloma cells were cultured with 0.5 μM of PHI for 48 or 96 hours. The cellular DNA content was determined by flow cytometry. Values are means +/- SD from 3 independent experiments. Open bar, G1 phase; Black bar, G2M phase; Grid bar, S phase.

To evaluate the effects of PHI on the growth of myeloma cells, the multiple myeloma cell line RPMI8226 was exposed to PHI at various concentrations. PHI caused a concentration- and time-dependent growth inhibition in these cultures. A significant growth inhibitory effect could be achieved with PHI at a concentration as low as 0.1 μM (Figure 1A). A 37.1% decrease of cell density was observed when 0.1 μM PHI was present in the cell culture. The cell proliferation was further reduced to 50% of that of control with PHI concentration at 0.5 μM. When the cells were exposed to PHI at the concentration of 5 μM, the culture became static. Morphological changes were also observed after exposure of RPMI 8226 cells to PHI. The cells appeared to have condensed chromatin, plasma membrane blebbing and cell shrinkage, characteristics of apoptosis (data not shown). The apoptosis was also confirmed by the significant increase of DNA strand breaks as measured by the TUNEL method (data not shown).

To study the effects of PHI on the cell cycle progression, the distribution of cells in different cell cycle phases was analyzed by flowcytometry. Figure 1B reveals a clear decrease of the replicating cells in S- and G2M-phases after PHI exposure for 48 hours at 0.5 μM (Figure 1B). The proportion of S- plus G2M-phases after 96 hr was decreased to 23.9%, as compared to 80.3% in the control cultures, indicating an approximately 3-fold reduction in cell proliferation. Along with the decrease of S- and G2M-phase cells, the cells in G1 phases showed a concomitant increase (Figure 1B). This is consistent with a G1-phase arrest.

### PHI induces p16 DNA hypomethylation

**Figure 2:**
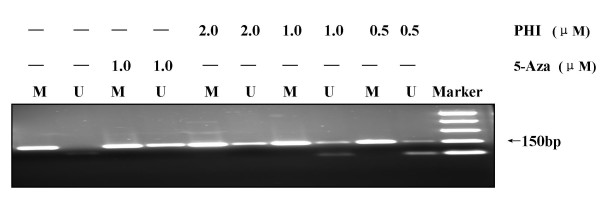
**PHI induces p16 hypomethylation in RPMI8226 myeloma cells**. The myeloma cells were cultured with or without phenylhexyl isothiocyanate (PHI) at varying concentrations for 10 days. Cellular DNA was extracted and bisulfite-converted as described in Material and Methods. Methylation-specific PCR was performed using primers specific for methylated and unmethylated DNA forms of p16, respectively. The PCR product was visualized after agarose gel electrophoresis. Decitabine (5-aza) was used as a positive control for DNA hypomethylation. M, methylated p16 fragment; U, unmethylated p16 fragment. The 150 bp marker position was indicated.

To explore the potential mechanisms of cell growth inhibition by PHI, we examined the status of DNA methylation and protein expression of a tumor suppressor gene, p16. Decitabine (5-aza-2-deoxy-cytidine), a known inhibitor of DNA methylation, was used as a positive control. In myeloma cells, p16 is known to be inactivated, due to aberrant CpG island methylation [6,7,32]. The status of DNA methylation was measured by methylation- specific PCR. Figure 2 reveals that the untreated cells have only the methylated form of p16, and there was no detectable level of the unmethylated form of p16. After exposure of the cells to PHI at 0.5 μM for 10 days, the unmethylated form became detectable, similar to that mediated by decitabine. There appeared to be an increase of hypomethylation of p16 when cells were exposed to higher concentrations of PHI (Fig. 2). The results suggested that PHI induced p16 hypomethylation in a concentration-dependent manner.

### PHI inhibits histone deacetylation

**Figure 3:**
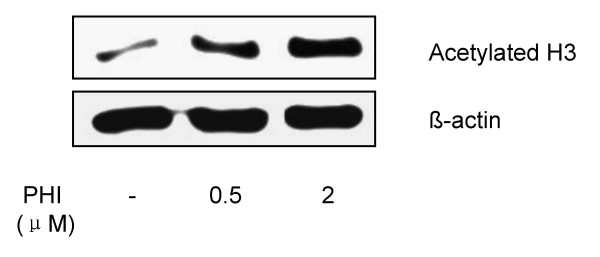
**PHI inhibits histone deacetylation in RPMI8226 myeloma cells**. The RPMI8226 myeloma cells were cultured with two different concentrations of PHI for 72 hours. The proteins were extracted from the cell lysates. The status of histone H3 acetylation was determined by Western blot as described in Material and Methods. β-actin level in the same blot was used as an internal loading control for protein amount.

We have previously shown that PHI can inhibit the histone deacetylase and induces histone hyperacetylation in HL-60 leukemia cells and prostate cancer cells [29-31]. The status of histone acetylation was examined in RPMI8226 myeloma cells after exposure to PHI. The acetylation of histone H3 was significantly increased in a concentration- dependent manner (Fig 3).

### PHI inhibits IL-6 receptor expression and reactivates p21 expression

**Figure 4:**
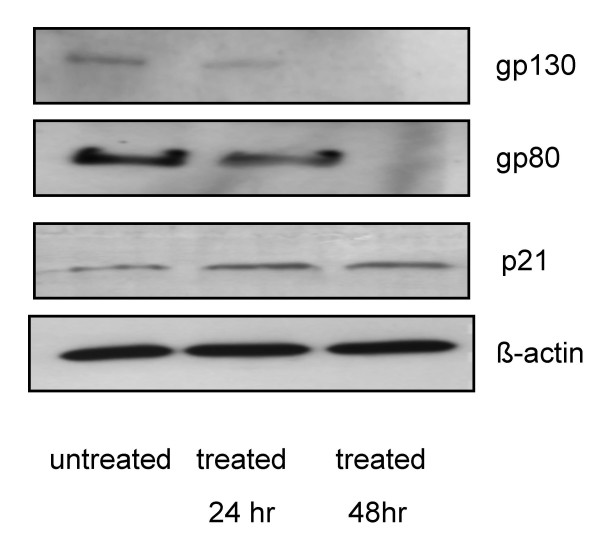
**PHI inhibits IL-6 receptor expression and reactivates p21 expression in RPMI8226 myeloma cells**. The RPMI8226 myeloma cells were cultured with 5 μM of PHI for 24 and 48 hours, respectively. The proteins were extracted from the cell lysates. The expression level of IL-6 receptor subunits, gp80 and gp130, as well as p21 protein were determined by Western blot analysis as described in Material and Methods. β-actin level in the same blot was used as an internal loading control for protein amount.

IL-6 mediated signaling pathway is known to be involved in myeloma pathogenesis, and is one of the mechanisms of drug resistance of myeloma cells [2,11]. We examined the effects of PHI on the expression of IL-6 receptors in RPMI8226 cells. PHI mediated a significant decrease of the expression of IL-6 receptor subunits gp80 and gp130 (Fig. 4). The expression of both receptor subunits was significantly reduced after 24 hr exposure to 5 μM PHI, and nearly diminished by 48 hrs. There was also a time-dependent increase of the level of p21 protein expression after PHI treatment (Fig.4). The reactivation of p21 expression by PHI has been demonstrated in our laboratory in other cell lines [29,30].

### PHI inhibits production of VEGF from myeloma cells

**Figure 5:**
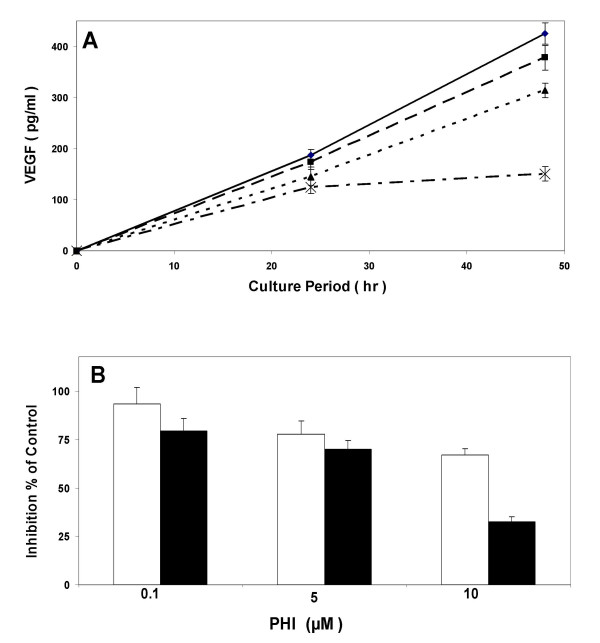
**PHI inhibits production of VEGF in RPMI8226 myeloma cells**. (A). The cells were incubated with varying concentration of PHI for 24 and 48 hours, respectively. VEGF production was determined as described in the Material and Methods. Diamond (♦), control; Square (■), 0.1 μM; Triangle (), 5 μM; Cross (x), 10 μM. (B). Percent inhibition of VEGF production as compared with the control culture was calculated from the above experiments. Values are means +/- SD from 3 independent experiments. Open bar, 24 hours; Black bar, 48 hours.

Angiogenesis has been considered as an important factor for the post-initiation and progression of myeloma, i.e. the metastasis of the tumor cells to the skeleton. We therefore analyzed the effects of PHI on the production of VEGF from the myeloma cell line. Figure 5 shows that PHI caused the suppression of VEGF production in a concentration- and time-dependent manner. At 10 μM, a significant 30% reduction of VEGF production was observed after 24 hr exposure, as compared to control cultures (*p *< 0.05). By 48 hrs, there was an approximately 70% reduction (*p *< 0.05).

### PHI induces disruption of mitochondrial membrane potential

**Figure 6:**
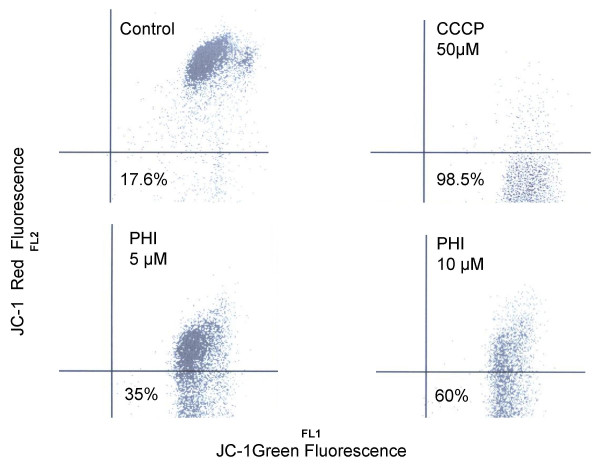
**PHI causes disruption of mitochondrial membrane potential in RPMI8226 myeloma cells**. The myeloma cells were treated with 5 μM and 10 μM of PHI, respectively for 48 hours. The cells were then stained with JC-1 dye. The mitochondrial membrane potential was measured by flowcytometry as described in the Material and Methods. The shift-down of fluorescence from Red to Green indicates the collapse of mitochondrial membrane potential. CCCP was used as a positive control for the disruption of mitochondrial membrane potential. The percent of cells with the disruption of mitochondrial membrane potential was indicated.

Previously we have demonstrated that PHI induced apoptosis through the activation of caspase-9, which often is the result of disruption of mitochondrial membrane potential. The latter led to the subsequent release of the effector molecules for apoptosis [35]. To further characterize the mechanism of apoptosis in the myeloma cells induced by PHI, we examined the status of mitochondrial membrane potential by a flowcytometric method after staining with JC-1, a dye that forms a color aggregate depending on the membrane potential. CCCP, a known agent that can cause disruption of mitochondrial membrane potential, was used as a positive control. Exposure of RPMI8226 cells to PHI caused a concentration-dependent shift from red fluorescence to green fluorescence, indicating the disruption of mitochondrial membrane potential (Fig. 6). There was a two fold increase in the number of cells with the disruption of mitochondrial membrane potential when 5 μM PHI was present for 48 hours. A 3.4-fold increase was observed when 10 μM PHI was present for 48 hours. The results indicated that PHI- induced apoptosis involves mitochondria.

## Discussion

This study demonstrated that PHI can inhibit the proliferation of the myeloma cell line RPMI8226 and induce apoptosis in a concentration as low as 0.5 μM. Most of the cells became apoptotic at a PHI concentration of 5 μM. These concentrations are lower than that required for growth inhibition and apoptosis in HL60 leukemia cells and prostate cancer cells that we have reported [29-31]. At the above concentration we have shown that PHI was nontoxic to normal human blood mononuclear cells [29]. We have initiated a protocol to investigate whether PHI has activities on myeloma cells obtained directly from patients.

In agreement with previous reports, p16 was found to be hypermethylated in RPMI8226 cells [7,32]. PHI induced hypomethylation of p16 at a concentration-dependent manner. The hypomethylation was comparable to that induced by decitabine, one of the two hypomethylating agents that have been approved for the therapy of myelodysplastic syndrome [36,37]. The reactivation of the tumor suppressor gene p16 may at least in part be responsible for the G1 arrest and apoptosis of RPMI8226 myeloma cells. There could be other genes and pathways that are activated by PHI-induced hypomethylation, because hypermethylation of tumor suppressor genes, such as SOCS-1, p16, E-cadherin, DAP kinase, MGMT, were frequently detected in myeloma cell lines as well as in clinical specimens from patients with plasma cell disorders [4,7]. The demethylation of p16 induced by PHI may involve DNA methyltransferase, which is being currently investigated.

Several HDAC inhibitors have been examined as a new class of potential drugs for treating myeloma [19,24]. We have recently reported that PHI is an inhibitor of HDAC and can induce selective histone acetylation and methylation changes in human leukemia cells [29,30]. The current study showed that PHI induced histone H3 hyperacetylation and p16 promoter hypomethylation. These findings suggest that PHI has dual effects of epigenetic modulation on both DNA and chromatin. The dual effects on DNA and chromatin from a single agent may provide a synergistic effect in reactivating p16 and other tumor suppressor genes. DNA methylation and histone deacetylation are linked in their actions for silencing gene expression. The methylated CpG-binding protein (MeCP2) recruits HDACs to specific promoter regions that induce the formation of repressive chromatin structure and transcription repression [32]. It would be interesting to study whether the combination of PHI and azacytidine has synergistic activity toward inhibition of myeloma cells. Clinical trials using a combination of HDAC inhibitors and hypomethylating agents are already underway for the therapy of patients with leukemia and myelodysplasia [38].

It was previously shown that when two HDAC inhibitors, SAHA and TSA, were combined, the cytotoxic effects on multiple myeloma were enhanced, and apoptosis was in part due to the disruption of mitochondrial membrane potential by the HDAC inhibitors [35]. The current study was in agreement with the above report. The combination of PHI and SAHA may enhance even more the cytotoxic effects on the myeloma cells due to the fact that PHI induces DNA hypomethylation as well as histone hyperacetylation.

IL-6 and IL-6 receptor- mediated signaling pathway is critical for the survival and proliferation of myeloma cells [1]. Angiogenesis inhibitors, thalidomide and lenalidomide, are new agents in the therapy of MM [1]. This study showed that PHI can inhibit the expression of IL-6 receptors and also reduce the cytokine VEGF produced by the RPMI 8226 myeloma cells. The latter corroborates a previous report of another HDAC inhibitor, valproic acid, that caused inhibition of VEGF production by RPMI8226 cells [26]. These results indicate that PHI targets several critical processes of myeloma survival and proliferation. One limit of this study is that it focused on one cell line, RPMI8226. Further experiments in more myeloma cell lines and in vivo animal studies would give further insights into the mechanisms and activities of PHI on myeloma cells. More studies are needed to further characterize this promising modulator of epigenetic processes for its potential in clinical applications.

## Conclusion

For the first time PHI was shown to induce both p16 hypomethylation and histone H3 hyperacetylation. We conclude that PHI has dual epigenetic effects on p16 hypomethylation and histone hyperacetylation in myeloma cells and targets several critical processes of myeloma proliferation.

## Authors' contributions

QL carried out cell cultures and participated in all assays and drafted the manuscript, XL performed p16 methylation analysis, MYL was actively involved in methylation analysis, JF, XZ, and RG were involved in western blot assays, JC, and DL were actively involved in concept design, coordination, data analysis, drafting and critically revising the manuscript.
